# Training the Next Generation of Academic Global Neurosurgeons: Experience of the Association of Future African Neurosurgeons

**DOI:** 10.3389/fsurg.2021.631912

**Published:** 2021-05-28

**Authors:** Ulrick Sidney Kanmounye, Yvan Zolo, Stéphane Nguembu, Francklin Tétinou, Lorraine Arabang Sebopelo, Geneviève Endalle, Dawin Sichimba, Régis Takoukam, Nathalie Ghomsi, Desmond T. Jumbam

**Affiliations:** ^1^Research Department, Association of Future African Neurosurgeons, Yaounde, Cameroon; ^2^Faculty of Health Sciences, University of Buea, Buea, Cameroon; ^3^Faculty of Medicine, Higher Institute of Health Sciences, Université des Montagnes, Bangangté, Cameroon; ^4^Faculty of Medicine, University of Botswana, Gaborone, Botswana; ^5^School of Medicine, Copperbelt University, Ndola, Zambia; ^6^Department of Neurosurgery, Felix-Houphouet Boigny University, Abidjan, Côte d'Ivoire; ^7^Operation Smile Ghana, Accra, Ghana

**Keywords:** Africa, capacity building, global neurosurgery, neurosurgery, research

## Abstract

**Introduction:** Although the past decade has seen a substantial increase in African neurosurgeons' academic productivity, productivity remains low compared to their colleagues from other regions. Aspiring neurosurgeons can contribute to the academic neurosurgery workforce by taking care of less technical and time-consuming research tasks. Fortunately, global neurosurgery institutions have also made efforts to increase research exposure and scholarly output in academic global neurosurgery. The Association of Future African Neurosurgeons (AFAN) created a research incubator for aspiring academic global neurosurgeons in Africa to provide enrollees with mentorship, skills, and experience. This study assesses and reports the activities and results of the research incubator.

**Methods:** Aspiring academic global neurosurgeons were enrolled in the AFAN Research Incubator Program (ARIP), whose primary objective was to provide enrollees with foundational skills in all aspects of the research cycle. ARIP enrollees participated in didactic and practical activities with the aim of publishing ≥1 article and presenting ≥1 abstracts at international conferences in one year.

**Results:** Fifteen AFAN members aged 25.0 ± 3.0 years enrolled in ARIP: 7 (46.7%) medical students, 4 (26.7%) general practitioners, and 4 (26.7%) residents. Eleven (73.3%) were male, 6 (40.0%) were from Cameroon and 6 (40.0%) had no previous research experience. Two (13.3%) enrollees dropped out. ARIP enrollees published a total of 28 articles, and enrollees published a median of 1.0 (IQR = 2) first-author articles on neurosurgical system strengthening. Additionally, ARIP enrollees presented 20 abstracts with a median of one abstract (IQR = 3.0).

**Conclusion:** South-South research collaborations like ARIP can contribute to improving global neurosurgery research capacity and output. These collaborations can set up the foundations for robust research in low- and middle-income countries.

## Introduction

Despite substantial increases over the past decade, African neurosurgeons' academic productivity remains low compared to their colleagues from other regions. More than one-third of African countries have no peer-reviewed neurosurgery articles, and the median number of articles for African countries with peer-reviewed articles is six publications (1). The low academic output is primarily due to a workforce shortage, and for the few neurosurgeons available, the patient workload is an impediment to research (2–4). Other barriers include a lack of exposure, lack of protected research time, limited access to articles, and lack of mentorship (5, 6). For example, more than 60% of aspiring African neurosurgeons do not have a mentor and have never presented an abstract at a conference, participated in a journal club, or contributed to a manuscript (7). One reason for the lack of exposure is that academic neurosurgeons do not have time for research activities (3, 4). Aspiring neurosurgeons can contribute to the academic neurosurgery workforce by taking care of less technical and time-consuming research tasks, creating time for academic neurosurgeons to do more research. The early involvement of aspiring neurosurgeons in research activities equally benefits them because it increases their exposure and skills.

Global neurosurgery institutions have made efforts to increase research exposure and scholarly output in academic global neurosurgery. For example, the World Federation of Neurosurgical Societies' Global Neurosurgery Committee has set-up a mentorship program and funding mechanisms (8). to promote research in low- and middle-income countries, no study, to our knowledge, has described a formal capacity-building initiative aiming to increase academic global neurosurgery exposure among aspiring neurosurgeons (5, 6).

The Association of Future African Neurosurgeons (AFAN), a 460-member neurosurgery interest group, created a research incubator for aspiring academic global neurosurgeons in Africa to provide enrollees mentorship, skills, and experience. This study aimed to assess and report the activities and results of the research incubator.

## Methods

**Figure 1:**
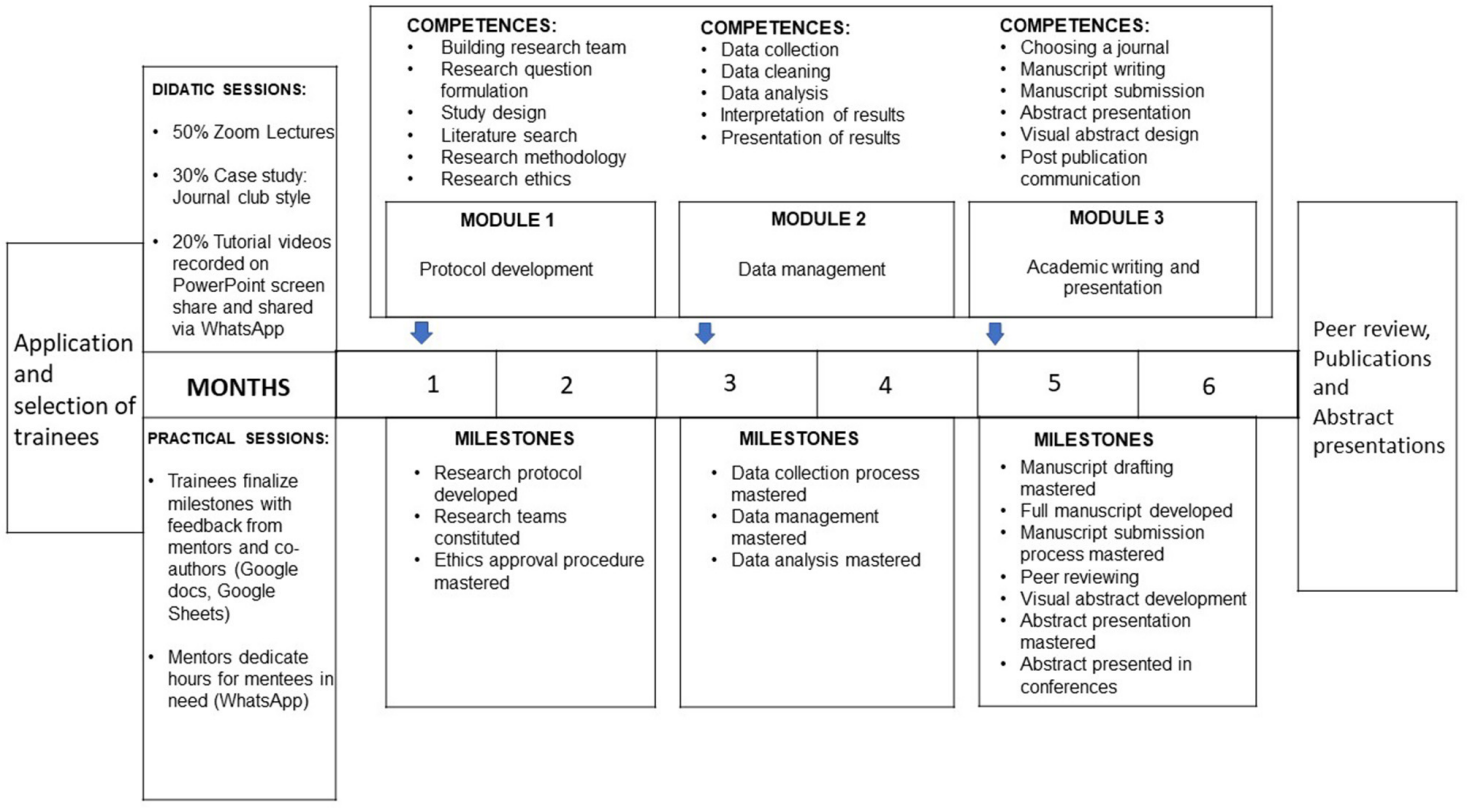
The Association of Future African Neurosurgeons Research Incubator Program structure.

On August 29, 2019, aspiring academic global neurosurgeons (medical students and general practitioners) were enrolled in the AFAN Research Incubator Program (ARIP) (Figure 1). ARIP was organized and led by the first author (USK), an experienced global neurosurgery researcher. Monthly lectures and journal clubs were organized on the first and second Saturday of each month at 5 p.m. GMT on Zoom (Zoom Inc., California, USA). The videos were recorded and shared with the enrollees for offline viewing. The authors obtained institutional review board approval before starting the project.

### ARIP Description

The primary objective of ARIP was to provide enrollees with foundational skills in all aspects of the research cycle: from study design to post-publication communication. The didactic courses were followed by practical exercises during which enrollees had to contribute significantly to a study.

Enrollees had to contribute significantly to five manuscripts, submit at least one article as a first-author, present at least one abstract at an international conference, design more than two visual abstracts, create an ORCiD account, create a ResearchGate account, and curate their social media accounts over 1-year.

The first author taught 28 90-min-lectures (60-min presentations and 30-min discussions). At the end of the lectures, each enrollee developed and proposed an idea for their first-author paper. Priority for first-author papers was given to studies involving human subjects if the first author was in their final year of medical school or residency and reviews (systematic, scoping, and narrative) if the first-author was not in their final year. This choice was made to avoid delays and costs related to institutional review board approvals, given that participants in their final year could get ethical approval easily.

**Figure 2:**
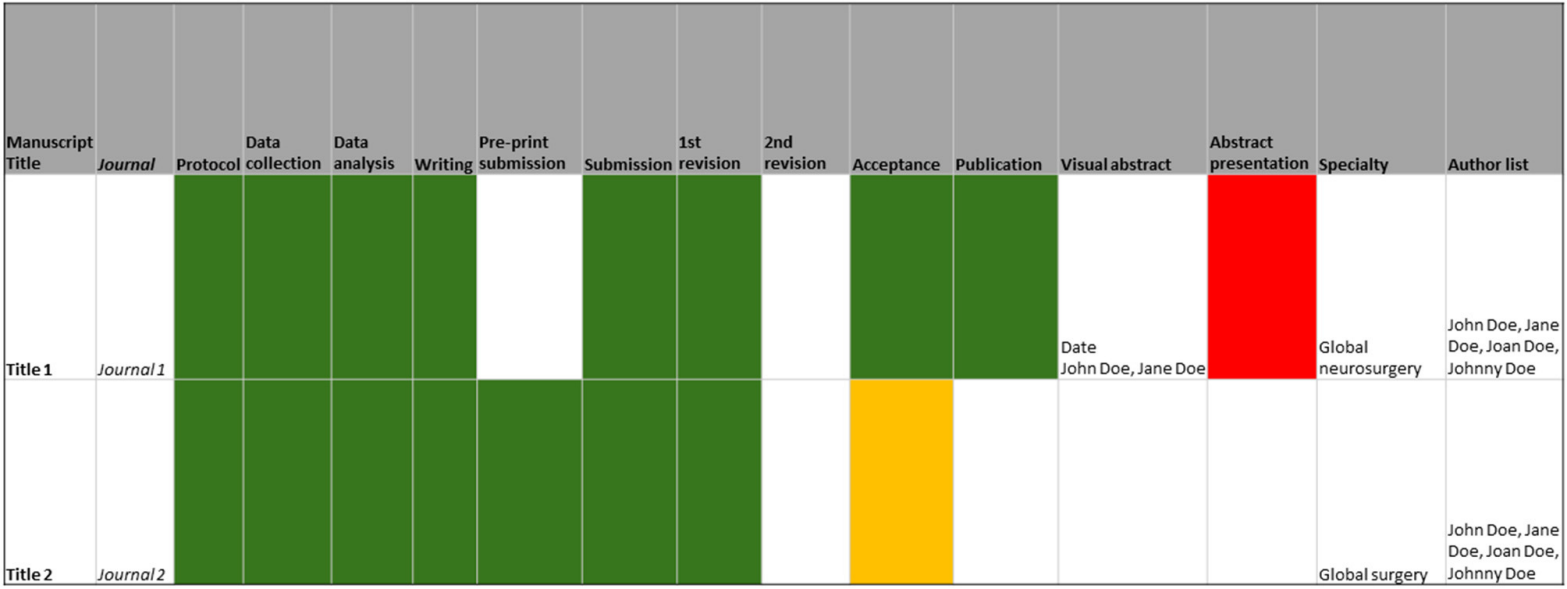
Research track sheet showing checkpoints of the research cycle with completion and deadlines. Completion is color coded: green (completed task), orange (uncompleted task and deadline past), red (major setback), and white (previous task has not been completed).

All research projects were logged in Google Sheets (Google Inc., California, USA) using a traffic light coding system along with target journals, target conferences, deadlines, and co-author lists (Figure 2). Priority was given to open-access journals without article processing charges and virtual conferences to maximize visibility and decrease expenses. The manuscripts were written on Google Docs (Google Inc., California, USA) and co-authors contributed using the suggestion mode. The online document history function was used to quantify the contributions of co-authors and to determine authorship positions. This was chosen to ensure transparency and accountability. Each research team held a monthly meeting with the first author during the study period. Day-to-day communications were done on WhatsApp (WhatsApp Inc., California, USA).

All abstracts accepted at conferences were first presented internally and reviewed by other ARIP enrollees before being presented at international conferences. Also, English and French visual abstracts were designed for all published articles and disseminated on AFAN and via the co-authors' social media handles.

### Statistical Analysis

Summary descriptive statistics were generated (gender, age, number of peer-reviewed articles pre-ARIP, number of peer-reviewed articles post-ARIP, and number of abstracts post-ARIP). A Wilcoxon signed-rank test was used to evaluate the difference in scholarly output before and after ARIP, and the *P*-value was considered statistically significant when <0.05.

## Results

Fifteen AFAN members aged 25.0 ± 3.0 years enrolled in ARIP: 7 (46.7%) medical students, 4 (26.7%) general practitioners, and 4 (26.7%) residents. Eleven (73.3%) were male, 6 (40.0%) were from Cameroon and 6 (40.0%) had no previous research experience (Table 1). Two (13.3%) enrollees dropped out (ID: 1 and 13).

**Table 1:** Socio-demographic data of the AFAN research incubator project.

**ID**	**Sex**	**Age**	**Total articles**	**First author articles**	**Abstracts**	**Profession**	**Country**	**Previous research experience**
1	M	25	8	0	3	GP	Cameroon	Thesis
2	F	27	15	2	2	Resident	Côte d'Ivoire	Thesis
3	M	26	15	5	3	GP	Cameroon	Thesis
4	M	26	22	3	5	GP	Cameroon	Thesis
5	M	24	18	3	5	Student	Cameroon	None
6	M	26	10	2	1	Resident	Côte d'Ivoire	Thesis
7	M	24	3	2	1	GP	DR Congo	Thesis
8	M	24	6	0	0	Student	DR Congo	None
9	M	33	8	1	0	Resident	Zimbabwe	Thesis and 4 articles
10	M	28	6	0	1	Resident	Morocco	Thesis and 5 articles
11	F	22	9	0	0	Student	Cameroon	None
12	M	23	10	2	3	Student	Zambia	1 article
13	F	20	3	0	0	Student	Zambia	None
14	M	24	4	0	0	Student	Cameroon	None
15	F	23	6	1	3	Student	Botswana	None

*GP, general practitioner*.

ARIP enrollees published a total of 28 articles and a median of 1.0 (IQR = 2) first-author articles. The increase in peer-reviewed publications was statistically significant (post-ARIP median = 8.0, IQR = 8.0 articles vs. pre-ARIP median = 0.0 articles; *P* = 0.01). Of note, the article titled *Barriers to the management of non-traumatic neurosurgical diseases at 2 Cameroonian neurosurgical centers: A cross-sectional study* was selected as an editor's choice in *World Neurosurgery* (9). The publications covered all the aspects of a neurosurgical system: workforce and infrastructure (10), finance, governance, information management (11), and service delivery (12).

ARIP enrollees presented 20 abstracts, of which 5 were presented at the *World Federation of Neurosurgical Societies Global Neurosurgery 2020 Conference*, and 3 were presented at *The Neurology and Neurosurgery Interest Group - Society of British Neurological Surgeons Conference* (Table 2). The enrollees presented a median of one abstract (IQR = 3.0).

**Table 2:** Scholarly output of the AFAN research incubator program.

**Title**	**Journal or conference**
**Manuscripts**	
1. Exploring the knowledge and attitudes of Cameroonian medical students toward global surgery: a web-based survey	PLoS ONE
2. Global neurosurgery: implications for low- and middle-income countries. The case of Cameroon	Iranian Journal of Neurosurgery
3. The role of young and future neurosurgeons in global neurosurgery: perspectives from the Association of Future African Neurosurgeons	Journal of Neurosciences in Rural Practice
4. Systemic disorders and the prognosis of stroke in Congolese patients	Ghana Medical Journal
5. Prehospital conditions and outcomes following craniotomy for traumatic brain injury performed within 72 h in Central Cameroon Cameroon: a cross-sectional study	World Neurosurgery
6. Qu'est-ce que la chirurgie globale et quel est le rôle des pays francophones dans la chirurgie globale ?	Pan African Medical Journal Clinical Medicine
7. Planning to succeed: career development resources for future African neurosurgeons	ECAJS Journal
8. Barriers to the management of non-traumatic neurosurgical diseases at two Cameroonian neurosurgical Centers: a cross sectional study	World Neurosurgery
9. Systematic review of patient attitudes toward neurosurgery in low- and middle-income countries	Neurology India
10. Hierarchy of scientific evidence and thematic analysis of African neurosurgery research – A scoping review and bibliometric analysis	Interdisciplinary Neurosurgery
11. African neurosurgery research: a scientometric analysis of the top 115 most cited articles	Interdisciplinary Neurosurgery
12. COVID-19 and neurosurgical education in Africa: making lemonade from lemons	World Neurosurgery
13. Advancing medical research in sub-Saharan Africa: barriers, facilitators, and proposed solutions	Pan African Medical Journal Clinical Medicine
14. How can African medical researchers use social media to their advantage? – Pearls and pitfalls	Pan African Medical Journal Clinical Medicine
15. Systematic review and bibliometric analysis of African anesthesia and critical care medicine research Part I: contributions and hierarchy of evidence	BMC Anesthesiology
16. Systematic review and bibliometric analysis of African anesthesia and critical care medicine research Part II: a scientometric analysis of the 116 most cited articles	BMC Anesthesiology
17. Understanding the motivations, needs, and challenges faced by aspiring neurosurgeons in Africa: an E-survey	British Journal of Neurosurgery
18. Bibliometric analysis of the 200 most cited articles in World Neurosurgery	World Neurosurgery
19. Spontaneous subdural hematoma in a third-trimester gravid patient: a case report	Interdisciplinary Neurosurgery: Advanced Techniques and Case Management
20. Pediatric TBI in Zimbabwe: a Prospective Cohort Study	Romanian Journal of Neurology
21. Increasing neurosurgery interest in Africa: an analysis of the Association of Future African Neurosurgeons' social media handles	International Journal of Medical Students
22. Mapping global neurosurgery research collaboratives: a social network analysis of the 50 most cited articles	Neurosurgery Open
23. Schizencephaly associated with blindness and deafness in a 10-month old infant: a case report and literature review	Ghana Medical Journal
24. Barriers and Facilitators of Research in Cameroon (Part I) - An e-survey of physicians	Pan African Medical Journal Clinical Medicine
25. Barriers and Facilitators of Research in Cameroon (Part II) - An e-survey of medical students	Pan African Medical Journal Clinical Medicine
26. Comorbidities associated with pediatric epilepsy at a Cameroonian tertiary teaching hospital: a cross-sectional study	Pan African Medical Journal Clinical Medicine
27. Factors associated with adverse outcomes in Cameroonian patients with traumatic brain Injury: a Cross-Sectional Study	Emergency Medicine International
28. Management of Skull Base Fractures in Cameroon: a multi-institutional cross-sectional study	Emergency Medicine International
**Abstracts**	
1. Global neurosurgery in Sub-Saharan Africa: estimating the neurosurgical workforce and infrastructural capacities in Cameroon	InciSioN Global Surgery Symposium 2020
2. Global surgery in Cameroon: evaluating the knowledge and attitudes of medical students toward global surgery	InciSioN Global Surgery Symposium 2020
3. Epidemiology of neurosurgical tumors at two reference centers in Cameroon	Multinational Association of Supportive Care in Cancer 2020
4. The burden of direct medical expenditures for epilepsy care among Congolese patients: a single-center study	World Federation of Neurosurgical Societies Global Neurosurgery 2020 Conference
5. African neurosurgery research (Part I): hierarchy of scientific evidence and thematic analysis	World Federation of Neurosurgical Societies Global Neurosurgery 2020 Conference
6. African neurosurgery research (Part II): a scientometric analysis of the top 115 most cited articles	World Federation of Neurosurgical Societies Global Neurosurgery 2020 Conference
7. Understanding the motivations, needs, and challenges faced by aspiring neurosurgeons in Africa: AN e-survey	World Federation of Neurosurgical Societies Global Neurosurgery 2020 Conference
8. Mapping Global Neurosurgery Research Collaboratives: a Social Network Analysis of the 50 Most Cited Articles	World Federation of Neurosurgical Societies Global Neurosurgery 2020 Conference
9. Paroxysmal sympathetic storm and the role of Beta-Blockers in moderate/severe head trauma: a scoping review.	National Research Collaborative Meeting 2020
10. Management and outcomes of pediatric intracranial suppurations in low- and middle-income countries: a scoping review	National Research Collaborative Meeting 2020
11. Trends in the indications and outcomes of cesarean section in Bukavu - A single-center cross-sectional study	National Research Collaborative Meeting 2020
12. Cerebral aneurysms in Africa: a scoping review	National Research Collaborative Meeting 2020
13. Systematic Review of Patient Attitudes Toward Neurosurgery in Low- and Middle-Income Countries	Bethune Round Table 2020
14. Acute myelopathy as a complication of schistosomiasis - A narrative review	Pan-African Organization for Health, Education and Research: Medical research and mentorship symposium
15. Developing Neurosurgical Research Interest Amongst Aspiring African Neurosurgeons during COVID19 Pandemic	Stanford Center for Innovation in Global Health: The 7th Annual Global Health Research Convening
16. Fostering neurosurgery interest among medical students and general practitioners in low- and middle-income countries: the Association of Future African Neurosurgeons Experience	The Neurology and Neurosurgery Interest
17. Cerebral Aneurysms in Africa: a literature review	Group - Society of British Neurological Surgeons Conference
18. Management of Basilar Skull Fractures in Cameroon: a multi-institutional cross-sectional study	The Neurology and Neurosurgery Interest Group - Society of British Neurological Surgeons Conference
19. Outcomes of Traumatic Brain Injury in Cameroon: a Cross Sectional Study	The Neurology and Neurosurgery Interest Group - Society of British Neurological Surgeons Conference
20. Decompressive craniectomy for severe traumatic brain injury in low- and middle-income countries: a retrospective cohort study.	The Young Continental Association of African Neurosurgical Societies Traumatic Brain Injury Symposium

All enrollees had designed ≥2 visual abstracts, created ORCiD and ResearchGate accounts and curated their Twitter and Facebook accounts. Eight (53.3%) enrollees had met the target set initially (≥five peer-reviewed articles, ≥one first-author article, ≥one abstract, ≥two visual abstracts, and curation of ORCiD, ResearchGate, and other social media accounts) at the time of publication.

The enrollees were asked about their experience, and the majority (86.7%) had positive feedback. Direct quotes from the enrollees are below:

- “[ARIP] is the best research experience I have had. The course director and my peers are very helpful and always available.”
- “I love that [ARIP] is goal-oriented and transparent. It motivates me to do my best.”
- “Publishing in respectable specialty journals and being awarded the Editor's choice is the consecration of our individual and team efforts.”

Participants identified project administration as the most challenging aspect of their research experience.

- “I find it hard to keep my co-authors motivated. I often have to do more work than was initially planned.”
- “I struggle to organize meetings and set deadlines.”

## Discussion

This global neurosurgery research capacity-building initiative is the first of its kind in Africa. We enrolled aspiring academic global neurosurgeons from all career levels. This program provides a framework for global neurosurgery research projects in low-resource settings and contributes to the attainment of research objectives set by the World Federation of Neurosurgical Societies' Global Neurosurgery Committee (8).

### Impact

ARIP has significantly increased the scholarly output of enrollees, and its publications and abstracts were featured in prestigious journals and conferences.

Locally-driven research is critical for the attainment of universal neurosurgical care. For this to happen, there must be a critical mass of experienced researchers within local academic institutions, locally-driven research agendas, stakeholder buy-in, and integration of research findings into high-level decision-making (13). ARIP is working to grow the local academic neurosurgeon workforce and map out research gaps through literature reviews. ARIP offers hands-on experience, provides medical students and physicians opportunities from multiple African countries, and is output-oriented. In addition, ARIP is a South-South partnership focused on research that is mindful of local realities - lack of funding, difficulties obtaining ethical clearance, and limited mentorship (6, 14). All these characteristics make ARIP a sustainable model for research capacity-building in Africa.

Rosenberg et al. (15) developed a similar research capacity-building project for emergency medicine physicians in Rwanda. At the end of the training, they presented six abstracts, published six manuscripts, and offered advanced-degree scholarships to 11 participants (15). While ARIP presented many more abstracts and published more articles, none of our participants were awarded an advanced degree scholarship. Advanced degrees further contribute to research capacity building and are correlated with increased scholarly output (16). Unfortunately, the cost of these degrees can be prohibitive even in low- and middle-income countries (17). Unlike ARIP, the Rwandan group had access to a substantial funding source i.e., a National Institutes of Health R21 grant, and could afford such an initiative. ARIP and organizations that do not have access to funding can still accompany their enrollees in their application to graduate schools and for full scholarships (Ex. Chevening, Fulbright, or Mastercard Africa).

AFAN has a strong presence across the continent (>7,000 likes and >15,000 weekly impressions on Facebook), enabling widespread dissemination of research findings, surveys, and opportunities. This has raised the profile of AFAN as an academic institution and has increased interest among prospective partners. Since starting ARIP, we have received invitations to collaborate from the Young African Neurosurgeons Committee, Global Neurosurgery Committee, Walter E. Dandy Neurosurgical Society, Neurology and Neurosurgery Interest Group. Also, interest in ARIP has increased significantly. Currently, we have enrolled 32 new fellows and recruited previous enrollees as trainers.

### Challenges and Limitations

We faced some challenges during ARIP. First, we lacked the resources to run more granular research. For example, we chose to avoid research involving human subjects to minimize the cost of institutional review board applications. Clinical research is an indispensable aspect of academic neurosurgery that evaluates the impact of systems-level changes on individual patients. Mindful of this, we intend to expand our research portfolio by collaborating with African neurosurgery centers. Next, our choice of open-access journals was restricted because we could not afford article processing charges. Often, one or more of our members was from a lower-middle or middle-income country, which meant that we were ineligible for a full waiver of article processing charges. Similarly, our choice of conferences for abstract submissions was limited because we could not afford registration fees. The transition of conferences to an online format due to COVID-19 from mid-2020 was a windfall for ARIP because most conferences waived registration fees. While we were able to accomplish a lot with limited resources, we acknowledge that some financial resources will be necessary to expand ARIP (15). To achieve this goal, AFAN has set up a grant development unit within the research department.

ARIP enrollees had to work on a tight schedule because the program was not integrated into their formal education. This meant that most projects took more time than necessary, especially during the exam period.

Despite the challenges faced, ARIP enrollees were satisfied with the program's quality and were willing to give back by training the new cohort of enrollees.

## Conclusion

In summary, ARIP aims to build skills and increase the exposure of aspiring academic global surgeons to increase the scholarly output in Africa. ARIP enrollees showed dedication, passion, and tremendous potential, suggesting that greater gains will be noted if this program is implemented in a more resourceful setting. We intend to expand this program and report on the progress of the first and subsequent cohorts (H-index, recruitment in academic institutions, successful grant applications, peer-review journal positions, and postgraduate education).

## Data Availability Statement

The original contributions presented in the study are included in the article/supplementary material, further inquiries can be directed to the corresponding author/s.

## Author Contributions

UK: conceptualization, methodology, investigation, visualization, and writing – original draft. YZ, SN, FT, LS, GE, DS, RT, and NG: writing - review and editing. DJ: supervision, methodology, validation, and writing - review and editing. All authors contributed to the article and approved the submitted version.

## Conflict of Interest

The authors declare that the research was conducted in the absence of any commercial or financial relationships that could be construed as a potential conflict of interest.
